# Microbiological safety of commercial canned and dry pet food products in Lebanon

**DOI:** 10.3389/fvets.2022.995184

**Published:** 2022-09-12

**Authors:** Mireille Serhan, Michella Hadid, Hani Dimassi, Maria Deghel, Hussein F. Hassan

**Affiliations:** ^1^Department of Nutritional Sciences, Faculty of Health Sciences, University of Balamand, Tripoli, Lebanon; ^2^Professional Trans-European Mobility Programme for University Studies (TEMPUS) Program in Food Science and Technology, Faculty of Arts and Sciences, University of Balamand, Tripoli, Lebanon; ^3^School of Pharmacy, Lebanese American University, Byblos, Lebanon; ^4^Nutrition Program, Department of Natural Sciences, School of Arts and Sciences, Lebanese American University, Byblos, Lebanon

**Keywords:** pet food, bacteria, microbiological quality, contamination, safety

## Abstract

Estimating the microbiological quality of pet food is essential in providing healthy and safe foods to pets. The aim of this study was to assess the microbiological safety of pet food marketed in Lebanon, namely cat and dog products. To the best of our knowledge, no studies have been conducted in Lebanon nor the Middle East region with reference to pet food quality. Lebanese market was screened and a total of 165 dry and canned pet food products were identified, collected and analyzed for their load of total aerobic microbial count, *Enterobacteriaceae* species, yeasts and molds, and for the presence of *Salmonella* and *Listeria* species. Dry pet food products had higher contamination level compared to canned ones. In terms of non-conformity to the European commission regulations, out of the 165 brands, 11 (7%) had a total aerobic microbial count above 10^6^ cfu/g, and 27 (16%) exceeded 3 × 10^2^ cfu/g as a maximum limit of presumptive *Enterobacteriaceae*. Among the dry brands, 8 out of 66 (12%) had a contamination level of yeasts and molds above 10^4^ cfu/g. Presumptive *Salmonella* spp. was detected in 68 (41%) and presumptive *Listeria* spp. in 106 (64%) of brands. These alarming results necessitates setting and monitoring microbiological standards for pet food in Lebanon. This study contributes as well to the building of a database for knowledge development regarding the potential contamination of pet food by the abovementioned microorganisms.

## Introduction

Nowadays, pet ownership, especially cats and dogs, is gradually increasing all over the world. Statistics reported that ~80 million European households (1) and 60% of the US houses (2) have at least one pet. This increase was well seen particularly during the COVID19 pandemic since pets are considered humans' companions, providing comfort and an easier way for the individual to cope and become healthier (3). Due to the rising number of pets, their food market is also evolving dynamically. Since 1940s, pet food have been manufactured in Europe and the USA based on animal feeds that were produced for livestock, and today most developed countries have pet food manufacturing plants (4).

It is necessary for pet food to be safe not only for pets, but also the pet owners and the environment. Besides the nutritional value of the food, microbiological safety is the main criterion in providing safe and healthy food (5). Research found that the percentage of pet owners feeding their pets commercial pet food constitute around 90% in both the United States and Australia because they consider these foods more convenient than preparing food themselves for their pets, since they meet all their nutritional needs (6) and they are less expensive (7). With reference to the changes in feeding practices between the years 2008 and 2018, despite the fact that most pets were fed heat-processed and commercial pet food, unlike previous years; feeding homemade or unconventional diets seems to be recently more prevalent than previously reported (8). This is because people think it is more affordable than buying processed feed at the retail store, think they are more palatable and have concerns about the nutritional value, in addition to the added chemicals and additives (6–8). The quality of wet and dry commercial pet food can worsen after purchase, even if they were sold healthy and safe (9).

There are several forms of hazards that can be found in pet food and can cause diseases to pets, including chemical hazards, like cyanuric acid (10), physical hazards like metal and other hard bodies (11). One of the most important aspects of pet food safety after processing is the microbiological quality including the criterion of the presence or absence of zoonotic agents. Some pathogens were previously detected in dry pet food samples, such as *Enterobacteriaceae* (12), *Salmonella* (13), *Listeria* species (14) and molds (15). Little research was done on the microbiological hazards occurring in canned food products. Instead, studies focused on the presence of pathogens in raw pet food such as *Salmonella* and *Enterobateriaceae* (16, 17).

Microorganisms present in pet food are not only a health risk for the pets, but also for the owners who have developed strong relationships with them. It has been shown that contaminated pet food can cause human illness through several routes like direct interaction with pet food or indirect contact between humans and objects that have come in contact with pet food. Some pets can carry the disease and be asymptomatic as well (18).

To ensure that pet food is safe, agencies such as the Food and Drug Administration (FDA), the United States Department of Agriculture and State feed agencies provide specific guidelines and regulations about pet food manufacture and labeling. According to Kukier et al. (19) about the microbiological quality of livestock feed, total aerobic microbial count (TAMC) should not surpass 10^6^ cfu/g. According to EU regulations No 142/2011 (20), dog chaws and processed pet food samples other than canned pet food samplesthat exceed 3 × 10^2^ cfu/g of *Enterobacteriaceae* are considered to be not satisfactory for the microbial hygiene. There are no regulations that specify the limit of *Listeria monocytogenes* species in pet food (21). It is assumed that *Listeria* species should meet the requirements defined for human foods. In other words, *Listeria* should be absent in 25 g of the feed (22). Furthermore, *Salmonella* should be absent in 25 g according to EU regulations (20) and the total number of yeasts and molds should not be >10^4^ cfu/g (23).

In Lebanon and the Middle East and North Africa (MENA) region, pet ownership has been on the rise recently, yet facts and figures are missing. To the best of our knowledge, no studies have been conducted in Lebanon nor the MENA region concerning microbiological pet food safety. Our study is the first of its-kind study assessing the occurrence of microbiological hazards in pet food marketed in Lebanon; as a result, the findings from this study can be used to provide a baseline data and to create awareness regarding pet food contamination in Lebanon.

## Materials and methods

### Sampling plan

With the aim of evaluating an exemplary selection of the different types of commercially prepared dogs and cats' foods available across Lebanon, 165 dog and cat food samples (99 commercially prepared canned products vs. 66 commercially prepared dry products) were collected from pet food shops and grocery stores located all over Lebanon, during Summer and Fall 2021. The samples'descriptions (pet food type, cat/dog, pet's age, protein source, grain/grain-free food and country of origin) are shown in Table 1. Samples collected were directly kept in their original package and only opened prior to analysis. All samples were tested twice for *Salmonella* and *Listeria* spp., for the number of *Enterobacteriaceae* spp. and for the total aerobic microbial count (TAMC). Only dry pet products were tested for the total yeasts and molds count (TYMC).

**Table 1:** Characteristics of commercially canned and dry pet food products (*n* = 165).

	***N***	**%**
**Type**		
Can	99	60
Dry	66	40
**Pet**		
Cat	81	49
Dog	84	51
**Pet's age^a^**		
NS^b^	19	11.5
Adult	33	20
Puppy or Kitten	14	8.5
**Protein source**		
Poultry	73	44
Meat	47	28.5
Fish	18	11
Poultry + fish	4	2.5
Poultry + meat	11	7
NS	12	7
**Include grain^a^**		
Grain	53	32
grain free	13	8
**Country**		
Europe	108	65.5
Asia	34	21
North/south America	17	10
Australia	1	0.5
NS	5	3

a , “Pet's age” and “include grain” categories were only specified for dry food products (Total 66).

b , Not Specified.

### Microbiological analysis

The preparation and dilutions of the samples were made according to standard ISO 6887–1:2017b−5 (24). From each sample, 25 g were transferred to nine times the volume (~225 ml) of buffered peptone water (Bio-rad, Marnes-la-Coquette, France) and homogenized for 1–2 min using a stomacher (BagMixer 400 W, interscience, France). A 10-fold serial dilution was prepared in 0.1% (v/v) peptone water. 0.1 mL of the mother solution (MS) and from each diluted mixture was separated by a pipette and moved to the petri dish. Some of the microorganisms were detected (*Salmonella* and *Listeria* species) and some were enumerated (TAMC, TYMC and *Enterobacteriaceae* species) after specific incubation time and temperature. Pet food samples were tested in accordance with standards that deal with microbiology of food and feeding stuffs (25):

#### Total aerobic microbial count (TAMC)

The sample was diluted as 10^−1^, 10^−2^, 10^−3^, 10^−4^, 10^−5^, and 10^−6^ and 0.1 ml of each dilution was spread on plate count agar (PCA) agar (HiMedia, India) for 42 h at 37°C ± 1°C. All the colonies grown on the plates were counted.

#### Enterobacteriaceae enumeration

The sample was diluted as 10^−1^, 10^−2^, 10^−3^ and 10^−4^ and 0.1 ml of each dilution was put in an empty petri dish where the Violet Red Bile Glucose (VRBG) agar (HiMedia Laboratories, India) was poured. The plates were incubated for 48 h at 37°C ± 1°C after agar solidification. Typical colonies grown on the abovementioned incubated plates, which have red color and red-pink halo were considered to be presumptive *Enterobacteriaceae* species and were counted.

#### Salmonella detection

*Salmonella* spp. isolation was conducted through a two-step enrichment procedure. After 24 h of incubation at 37°C ± 1°C in Buffered Peptone Water, 0.1 ml wasinoculated onto Rappaport-Vassiliadis RVS broth (Oxoid, USA) and 1 ml into tetrathionate broth (Oxoid, USA). Both enrichments were incubated at 42°C ± 1°C for 24 h and then plated into XLD (Difco, USA) and Salmonella Shigella Agar (CondoLab, Spain) and incubated at 37°C for 24 h. The appearance of black colonies after incubation at 37°C ± 1°C for 24 h suspects the presence of *Salmonella* species. All suspected species were selected and counted as presumptive *Salmonella* species without further confirmatory tests.

#### Listeria detection

After 24 h of incubation at 37°C ± 1°C in Buffered Peptone Water, 1 ml was inoculated into a tube containing 10 ml Frazer broth. The enrichment was incubated at 42°C ± 1°C for 24 h. A portion using a loop was taken from the broth and spread on the surface of Palcam agar (HiMedia, India). The appearance of black colonies after incubation at 37°C ± 1°C for 24 h implied that there was presence of *Listeria* species. All suspected species were selected and counted as presumptive Listeria species without further confimatory tests.

#### Yeasts and molds enumeration

The sample was diluted as 10^−1^, 10^−2^, 10^−3^ and 10^−4^ and 0.1 ml of each dilution was spread on Sabouraud agar (CondaLab, Spain) for 5 days at 25°C ± 1°C. The colonies that were grown on the plate were suspected to be yeasts and molds.

According to ISO 7218 (25), the presence of microorganisms and their quantity were analyzed. Microbial counts were expressed as the logarithm of colony forming units per gram of sample.

### Statistical analysis

Pet food products information and laboratory analysis results were coded and entered into SPSS V26 for further analysis. “Microorganisms results including concentrations of TAMC, Enterobacteriaceae and TYMC were regrouped as “Below the quantification limit (BQL),” “10^2^-<10^6^ (TAMC), 10^2^-<3 × 10^2^ (Enterobacteriaceae), 10^2^-<10^4^ (TYMC) cfu/g,” and “>10^6^ cfu/g.” All laboratory results and can food characteristics were summarized using frequency (N) and percentages (%). Bivariate analysis to determine the effect of can food characteristics on microorganisms' concentration were tested using the Pearson Chi-square. P-value below 5% were indicative of statistical significance.

## Results

“Microorganisms results including concentrations of TAMC, Enterobacteriaceae and TYMC were regrouped as “BQL,” “102-<106 (TAMC), 102-<3 × 102 (Enterobacteriaceae), 102-<104 (TYMC)cfu/g,” and “>106 cfu/g.” All 165 samples analyzed for TAMC ranged from BQL to above 3 × 10^7^ cfu/g. Among them, 51 (31%) samples had a contamination level above 10^4^ cfu/g, of which 11 (6.7%) recorded a contamination level above 10^6^ cfu/g. On the other hand, *Enterobacteriaceae* was detected in 50 (30%) samples. The load of *Enterobacteriaceae* ranged from BQL to 7 × 10^4^ cfu/g. *Salmonella* spp. was detected in 68 (41%) and *Listeria* spp. in 106 (64%) of the samples. Furthermore, 8 (12%) samples had a contamination level of TYMC above the limit, and all these samples contained at least one cereal (maize, wheat, rice and/or oats). The contamination level ranged from BQL to 3 × 10^4^ cfu/g.

The statistical results of the microbiological quality in both dry and canned pet food are shown in Tables 2–6. There was a significant correlation between the type of pet food (can/dry) and the level of contamination of each microorganism (*P* < 0.05) except for TYMC, since it is only tested on dry pet food. Another significant difference was found among pet food containing different cereals. The majority of the dry samples containing grains (87%) had a contamination level of TYMC below the limit.

**Table 2:** Microbiological results of Total Aerobic Microbial Count in dry and canned pet food.

**TAMC**	**BQL^a^**		**Below 10^6^ cfu/g**		**Above 10^6^ cfu/g**		***p*-value**
	***N***	**%**	***N***	**%**	***N***	**%**	
**Type**							
Can	0	0%	95	96%	4	4%	
Dry	2	3%	56	85%	8	12%	**0.029**
**Pet**							
Cat	2	2.5%	74	91%	5	6%	
dog	0	0%	77	92%	7	8%	0.310
**Pet's age**							
NS^b^	0	0%	15	79%	4	21%	
Adult	1	3%	29	88%	3	9%	
Puppy or kitten	1	7%	12	86%	1	7%	0.515
**Protein source**							
Poultry	2	3%	65	89%	6	8%	
Meat	0	0%	43	91.50%	4	8.50%	
Fish	0	0%	17	94%	1	6%	
Poultry + fish	0	0%	4	100%	0	0%	
Poultry + meat	0	0%	11	100%	0	0%	
NS	0	0%	11	92%	1	8%	0.944
**Presence of grain**							
Grain	0	0.00%	46	87%	7	13%	
Grain free	2	15%	10	77%	1	8%	**0.014**
**Country**							
Europe	2	2%	99	92%	7	6.50%	
Asia	0	0%	30	88%	4	12%	
North/south America	0	0%	16	94%	1	6%	
Australia	0	0%	1	100%	0	0%	
NS	0	0%	5	100%	0	0%	0.953

a , Below the Quantification Limit.

b , Not specified.

TAMC, total aerobic microbial count.

## Discussion

The list of biological hazards that might be found in pet food and that can cause diseases to animals if not monitored include *Salmonella, Listeria, Enterobacteriaceae* and yeasts and molds (26). According to Kim et al. (27), in order to ensure food safety and reduce food loss globally, monitoring food quality throughout the food supply chain and especially biological hazards is very important. The microbiological quality of meat for example depends on several factors, including the physiological status of the animal at slaughter, processing, the temperature and other conditions of storage and transportation (28). This study shows that dry and canned pet food products may harbor food-borne pathogens such as *Salmonella, Listeria, Enterobacteriaceae* and fungi, and pet owners should take serious precautions when handling pet food.

According to Tables 2–**5**, the number of dry samples had higher bacterial contamination than canned samples (*P* < 0.05). A deflection from good manufacturing practices (GMP) or cross-contamination from other sources are the main reasons for contamination with pathogens (29). After heat treatment, dry products are more likely to be contaminated with bacteria compared to canned products that are considered to be a safe alternative regarding biological hazards such as bacteria and parasites because cans are usually sterilized (30). It is suggested that dry foods, once opened, are stored for a long time since they contain a large amount of feed in contrast with cans which are usually consumed at once. Another important factor might be the poor barrier properties of dry pet food packaging and poor storage practices, especially that Lebanon has been witnessing in the last 2 years an unprecedented power crisis, resulting in absence of control for temperature and humidity during storage.

### Total aerobic microbial count

Microbial growth can make food less pleasant to eat (spoilage) and can make the consumer ill. Until today, no strict regulations have been applied concerning the maximum limits of bacterial and fungal contamination in pet food (21, 31). According to Kukier et al. (19) about the microbiological quality of livestock feed, total aerobic micribial count (TAMC) should not surpass 106 cfu/g.”

The pet food samples analyzed for TAMC in our study ranged between BQL to above 3 × 10^7^ cfu/g. A great variation was seen among samples from different manufacturers, and even among samples having the same manufacturer but different main ingredients or different target pet groups (data not shown). For instance, 51 (31%) of the samples had a TAMC contamination above 10^4^ cfu/g, of which 11 of the samples (7%) indicated a contamination level above 10^6^ cfu/g. A study conducted by Holda et al. (12) reported that 75% of the dry foods marketed in Poland have been contaminated, but with lower ranges: between 1.0 × 10^1^ and 2.7 × 10^2^ cfu/g. In contrast, the percentage was lower than the results of a study done by Kazimierska et al. (31) where 14% of the 36 commercial dry dog foods collected from the European market had a contamination level above 10^6^ cfu/g.

The unhygienic conditions in which animal feed is prepared, distributed and even stored in the house raise a question on the microbiological quality that is present in these foods, that might be transmitted to humans, and that might cause diseases to both humans and pets (32). In addition to spoiled raw material and bad distribution circumstances, the conditions that affect the multiplication and metabolism of microorganisms during storage are water, light, pH, nutrients, inhibitors, light, time and oxygen (33). For example, high temperature usually decreases the survival rate of the microorganism because of the denaturation of cellular components (34). Concerned authorities should put all pet food through labeling requirements such as nutrient content, ingredient list, product name and nutritional adequacy affirmations, with the ingredients being GRAS (generally recognized as safe), as defined by the association of American feed control officials for their use or approved as food additives (35). To add, according to Eirmann et al. (36), it is important for the pet food manufacturers to be a part of the association of American Feed Control (AAFCO) to ensure good manufacturing practices like proper storage and record keeping, and have a Hazard Analysis and Critical Control Points (HACCP) to eliminate the hazards as much as possible such as providing thermal treatment to destroy the pathogens.

Aside the significance between the type of pet food (dry/can) and the contamination level, dry samples containing a grain showed higher TAMC contamination compared to grain-free samples (*P* = 0.014). Cereals including wheat, maize, barley and rice are the most prevalent in the production of dry pet food, replaced by beet and potato in grain-free pet food production (37). Microorganisms that might be found in grains can be pathogenic bacteria like *Salmonella, E. coli* and *Bacillus cereus*, non-pathogenic bacteria like *Lactobacillaceae, Bacillaceae, Pseudomonadaceae* and *Micrococcaceae*, and mycotoxigenic fungi which are mostly *Penicillium, Fusarium, Helminthosporium, Aspergillus, Alternaria* and *Cladosporium* (37). Cereals can affect the number of TAMC, the quality of pet food and the health of pets consuming it.

### *Enterobacteriaceae* spp.

Presumptive *Enterobacteriaceae* was detected in 50 of the 165 samples analyzed (30%). According to EU regulations No 142/2011 (20), dog chaws and processed pet food samples other than canned pet food samples that exceed 3 × 10^2^ cfu/g of *Enterobacteriaceae* are considered to be not satisfactory for the microbial hygiene.”

The number of *Enterobacteriaceae* ranged between BQL to 7 × 10^4^ cfu/g. Of the 50 positive samples, 27 (16%) had levels above 3 × 10^2^ cfu/g. Our results indicated higher values compared to other studies. For example, Wojdat et al. (38) reported that 10% of the dry pet food samples collected across Poland and tested for *Enterobacteriaceae* had a contamination level above 3 × 10^2^ cfu/g. In contrast, Holda et al. (12) reported that 60% of the dog food samples tested in Poland were contaminated with *Enterobacteriaceae*. The occurrence of pathogenic bacteria from raw pet food was tested by Hellgren et al. (39), and it was found that *Enterobacteriaceae* was isolated from all the samples, and 60% exceeded the maximum level. Another study revealed that 72.5% of raw pet food samples available in Switzerland, did not meet the microbiological regulations set by the EU (40). The high contamination in raw food is normal because raw foods do not undergo heating and other processing techniques. This was shown in the screening of raw and non-raw pet food for the presence of extended-spectrum beta-lactamase producing *Enterobacteriaceae*, when the microorganism was isolated from 77.8% of the raw pet food and 0% from non-raw pet food (41). According to Carvalho et al. (42), pets, especially dogs, were shown to be an important source of multiresistant *E. coli* strains in the households, which can be transferred to humans through several routes, and cause serious health problems.

According to Takahashi et al. (43), food manufacturers consider *Enterobacteriaceae* a hygiene indicator. This explains that the presence of *Enterobacteriaceae* in pet food may indicate poor sanitation in the processing surroundings or improper processing. Some *Enterobacteriaceae* like *E. coli* and *Enterobacter* spp. can cause extraintestinal opportunistic infections in dogs like urogenital infections, which are infections of the kidneys, urethra, bladder and parts of the genital tract such as the uterus and the prostate, and can also cause meningitis, sepsis and surgical site infection (44).

With reference to Table 3, dry samples had more contamination with *Enterobacteriaceae* compared to canned samples (*P* < 0.01). Our results are contradictory to two studies conducted by Kukier et al. (19) and Kepińska-Pacelik et al. (21), which reported that wet pet food showed higher contamination level of *Enterobacteriaceae* than dry foods. This might be caused by the ability of survival of some *Enterobacteriaceae* in low moisture for a long period of time (45), and this was seen in the manufacturing of infant formulae where *Salmonella* and *Enterobacteriaceae* risks in the finished product are met on the dry part of the procedure (46). Also, grain containing dry pet food had higher *Enterobacteriaceae* contamination than grain-free foods (*P* = 0.010). According to a study conducted by Olstorpe et al. (47, 48), two *Enterobacteriaceae* species *Pantoea agglomerans* and *E. coli* can grow at low moisture content and on cereal grain. In addition, the country of origin of pet food samples was a source of significance (*p* = 0.004) in our study. Asian countries had the most *Enterobacteriaceae* contamination, after which comes the European countries. Some cans that were made in EU, were produced just for Lebanon, as per the label. These cans might be contaminated unlike the others that are produced not for a specific country. The lack of microbiological standards concerning the allowable quantity of microorganisms in pet food in Lebanon and poor controls on imports might be the reason, which in turn is a potential health risk for both pets and their owners.

**Table 3:** Microbiological results of presumptive *Enterobacteriaceae* in dry and canned pet food.

	**BQL^a^**		**<3 × 10^2^ cfu/g**		**Above 3 × 10^2^ cfu/g**		***p*-value**
	***N***	**%**	***N***	**%**	***N***	**%**	
**Type**							
Can	83	84%	8	8%	8	8%	
Dry	31	47 %	16	24%	19	29%	**<0.001**
**Pet**							
Cat	59	73%	7	9%	15	18.5%	
dog	55	65.5%	17	20%	12	14%	0.101
**Pet's age**							
NS^b^	13	68%	3	16%	3	16%	
Adult	11	33%	10	30%	12	36%	
Puppy or kitten	7	50%	3	21%	4	29%	0.194
**Protein source**							
Poultry	54	74%	7	10%	12	16%	
Meat	29	62%	11	23%	7	15%	
Fish	14	78%	2	11%	2	11%	
Poultry + fish	2	50%	1	25%	1	25%	
Poultry + meat	9	82%	1	9%	1	9%	
NS	6	50%	2	17%	4	33%	0.493
**Presence of grain**							
Grain	20	37%	15	28%	18	34%	
Grain free	11	85%	1	8%	1	8%	**0.010**
**Country**							
Europe	80	74%	13	12%	15	14%	
Asia	21	62%	4	12%	9	26.5%	
North/south America	12	71%	3	18%	2	12%	
Australia	1	100%	0	0%	0	0%	
NS	0	0%	4	80%	1	20%	**0.004**

a , Below the Quantification Limit.

b , Not specified.

### Presumptive *Salmonella* spp.

According to EU regulations (20), *Salmonella* should be absent in 25 g of product. In our study, *Salmonella* was detected in 68 (41%) of the total pet food samples. The incidence of *Salmonella* is very high compared to previous studies, and according to Table 4, higher number of dry samples had *Salmonella* contamination than canned samples (*P* < 0.01). The results are in contrast with a study conducted by D'aoust et al. (16), where no *Salmonella* contamination was found in all tested pet food samples in Poland. In addition, 0% of canned pet food products and only 0.96% of dry products tested in Poland were positive for *Salmonella* (17). In a study analyzing the prevalence of microbial organisms in pet food, it was observed that 8% of the tested samples were positive for *Salmonella* species, with all the feed being raw and only 1 dry (14). Hellgren et al. (39) noted the contamination of 7% of the raw meat-based products tested in Sweden and Yukawa et al. (49) observed an incidence of *Salmonella* of 2% of the dog treats collected in Japan. *Salmonella* may have originated from the meat of the animals it was derived from since it can colonize their intestines or be asymptomatically infected, or from the vegetables and spices used as additional ingredients to the feed (50). Pet food owners should be aware that bacteria like *Salmonella* is a zoonotic pathogen that can be transmitted from pets to humans. Dry dog and cat food from a certain manufacturer were linked to *Salmonella Schwarzengrund* outbreak where 79 cases were identified in the United States (13). According to Lambertini et al. (51), *Salmonella* can contaminate food ingredients during processing or its environment, inadequate heat treatments and recontamination after extrusion can also be the cause of *Salmonella* poisoning. When talking about Salmonellosis, diarrhea is the most common symptom, but usually clinical Salmonellosis is rare in dogs and cats and they can become carriers for a considerable amount of time (52). Some pets can carry the disease and be asymptomatic, and then transfer it to humans; however, this is rare in dogs and cats which can become carriers of the illness for a long-time infecting people when they are handling contaminated pet food or when they are in contact with cats or dogs. Even pets who are asymptomatically infected can shed *Salmonella* for 3 weeks and up to 3 months (53).

**Table 4:** Microbiological results of presumptive *Salmonella* spp. in dry and canned pet food.

	**Absent in 25 g^a^**		**Present in 25 g**		***p*-value**
	***N***	**%**	***N***	**%**	
**Type**					
Can	73	74%	26	26%	
Dry	24	36%	42	64%	**<0.001**
**Pet**					
Cat	49	61%	32	40%	
dog	48	57%	36	43%	0.662
**Pet's age**					
NS^b^	6	32%	13	68%	
Adult	12	36%	21	64%	
Puppy or kitten	6	43%	8	57%	0.801
**Protein source**					
Poultry	42	57.5%	31	42.5%	
Meat	29	62%	18	38%	
Fish	12	67%	6	33%	
Poultry + fish	1	25%	3	75%	
Poultry + meat	8	73%	3	27%	
NS	5	42%	7	58%	0.429
**Presence of grain**					
grain	15	28%	38	72%	
grain free	9	69%	4	31%	**0.006**
**Country**					
Europe	67	62%	41	38%	
Asia	20	59%	14	41%	
North/south America	10	59%	7	41%	
Australia	0	0%	1	100%	
NS	0	0%	5	100%	0.060

a , According to EU (142/2011) (20).

b , Not specified.

Moreover, *Salmonella* contamination was higher in grain containing pet foods than in grain-free food (p=0.006). According to Lauer et al. (54), *Salmonella* can survive a period of 52 weeks and *E. coli* above 44 weeks on wheat grain. Another study recorded *Salmonella* contamination of compounded feedstuffs containing cereal crops for livestock in the United Kingdom (55).

### Presumptive *Listeria* spp.

As for *Listeria*, it should also be absent in 25 g of the pet food or its contamination level must be <100 cfu/g (22). In our study, 106 out of 165 (64%) samples were positive for Listeria species. The level of contamination was too high compared to a previous study conducted by Nemser et al. (14), where 16% of the samples were tested positive for *Listeria monocytogenes* and 14% for other *Listeria monocytogenes* spp.). According to the Center of Veterinary Medicine (18), *Listeria* spp. can cause mild gastrointestinal signs, fever, muscle pain, breathing problems, pregnancy loss, and even death. After cats and dogs consume contaminated pet food, some of them do not show signs of Listeriosis, but they become carriers of *Listeria monocytogenes*, shed it in their stool and then spread it in the house or to the people in the household.

Along with the significant difference in *Listeria monocytogenes* between dry and canned foods, dogs and cats also showed a significant difference (*P* = 0.05) (Table 5). This can be attributed to the fact that cat and dog food do not include the same ingredients since cats are strictly carnivorous feeding on animal tissues to get all their nutritional requirements, consuming prey mainly high in proteins with moderate amounts of carbohydrates and minerals; however, dogs are omnivorous and can switch to eating plants and fruits in case of famine (56). According to the Center for Food Safety and Applied Nutrition (57), Listeria monocytogenes is not only found in refrigerated ready-to-eat foods like meat, dairy products, poultry and seafood, but also in produce harvested from soil, and can grow in refrigerated temperatures. This confirms the high prevalence of Listeria in cans, since cans, if opened, are immediately stored in the refrigerator. In addition, there was a significant correlation between grain containing food and contamination with Listeria (*P* < 0.01). As mentioned above, and according to literature, there was a significant correlation between grain containing food and occurrence of *Salmonella* and *Enterobacteriace*. No previous research has correlated the occurrence of *Listeria* and grain containing food.

**Table 5:** Microbiological results of presumptive *Listeria* spp. in dry and canned pet food.

	**Absent in 25 g^a^**	**Present in 25 g**	***p*-value**		
	***N***	**%**	***N***	**%**	
**Type**					
Can	45	45.5%	54	54.5%	
Dry	14	22%	52	79%	**0.010**
**Pet**					
Cat	35	43%	46	57%	
dog	24	29%	60	71%	**0.050**
**Pet's age**					
NS^a^	6	32%	13	68%	
Adult	7	21%	26	79%	
Puppy or kitten	1	7%	13	93%	0.237
**Protein source**					
Poultry	34	46%	39	53%	
Meat	16	34%	31	66%	
Fish	5	28%	13	72%	
Poultry + fish	1	25%	3	75%	
Poultry + meat	2	18%	9	82%	
NS	1	8%	11	92%	0.078
**Presence of grain**					
Grain	5	9%	48	91%	
Grain free	9	69%	4	31%	**<0.001**
**Country**					
Europe	44	41%	64	59%	
Asia	8	24%	26	77%	
North/south America	6	35%	11	65%	
Australia	1	100%	0	0%	
NS	0	0%	5	100%	0.093

a , Not specified.

**Table 6:** Microbiological results of total yeasts and molds count in dry pet food.

**TYMC**	**ND^a^**		**Acceptable/Below the limit (10^4^ cfu/g)^b^**		**Not Acceptable/Above the limit (10^4^ cfu/g)**		***p*-value**
	***N***	**%**	***N***	**%**	***N***	**%**	
**Type**							
Can	–	–	–	–	–	–	
Dry	32	49%	26	39%	8	12%	
**Pet**							
Cat	17	59%	11	38%	1	3%	
Dog	15	41%	15	41%	7	19%	0.114
**Pet's age**							
NS^c^	15	79%	2	11%	2	11%	
Adult	13	39%	16	49%	4	12%	
Puppy or kitten	4	29%	8	57%	2	14%	**0.024**
**Protein source**							
Poultry	17	50%	13	38%	4	12%	
Meat	6	50%	2	17%	4	33%	
Fish	0	0%	4	100%	0	0%	
Poultry + fish	3	75%	1	25%	0	0%	
Poultry + meat	0	0%	1	100%	0	0%	
NS	6	55%	5	46%	0	0%	0.085
**Presence of grain**							
Grain	23	43%	22	42%	8	15%	
Grain free	9	69%	4	31%	0	0%	0.157
**Country**							
Europe	14	40%	17	49%	4	11%	
Asia	5	46%	5	46%	1	9%	
North/south America	10	71%	3	21%	1	7%	
Australia	1	100%	0	0%	0	0.%	
NS	2	40%	1	20%	2	40%	0.321

a ,Not determined.

b , According to GMP (23).

c , Not specified.

TYMC, total yeast and mold count.

### Total yeasts and molds count

Yeasts and molds were tested only for dry pet food products since cereals are one of the most important ingredients of dry pet food that can be vectors of dangerous mycotoxins produced by molds, posing a health threat on pet lives as well as their owners (37). The total number of yeasts and molds should not be >10^4^ cfu/g (23). In this current study, the contamination level ranged from 0 to 3 × 10^4^ cfu/g. Among the samples, 8 (12%) had a contamination level above the limit, and all these samples contain at least one of type of cereals (maize, wheat, rice and/or oats). Previous studies have also detected yeasts and molds in pet food. For example, Wojdat et al. (38) found that 9% of the analyzed animal feeds were contaminated with fungi. Bueno et al. (58) noticed that all the commercial dry dog food samples tested were contaminated with yeasts and molds. Also, when evaluating the microbiological quality of pet food, Kazimierska et al. (31) reported the presence of molds in the analyzed samples ranging from 1 × 10^1^ to 1 × 10^5^ cfu/g. These results are in contrast with those reported by Holda et al. (12), who did not find any fungal contamination above 2 × 10^2^ cfu/g. Various foodborne molds and some yeasts might be toxic to animals and introduced *via* several routes because of their ability to produce mycotoxins. Molds do not always produce mycotoxins, as there are several factors that affect their formation like the presence or absence of inhibitors and nutrients, the weather conditions, geographic and seasonal factors, susceptibility of the crop, humidity, temperature, cultivation, harvesting as well as storage and transportation practices (59).

## Strengths and limitations

This study has two main limitations that need to be acknowledged. First, the microbiological safety of pet food marketed in Lebanon was evaluated using only classical methods of culture, without conducting further confirmatory tests. Second, only canned and dried pet food samples were collected and analyzed; raw meat-based diets for dogs were not included. As for the strengths of this study, and to the best of our knowledge, no previous research has been conducted in Lebanon nor the MENA region on assessing the microbiological quality of pet food. Therefore, our study is the first of its-kind study evaluating the occurrence of microbiological hazards in pet food marketed in the Lebanese market. To add, the evaluation of each of the sample type (canned/dry, cat/dog, age, protein source, grain/grain free, and country of origin) is considered another significant strength.

## Conclusion

The results reported from this study show the necessity to shed the light on the microbiological safety of pet food marketed in Lebanon, since 51 (31%) of the tested samples had TAMC contamination level above 10^4^ cfu/g, of which 11 (7%) had contamination above 10^6^ cfu/g. Moreover, 27 (16%) of the samples had a contamination level of *Enterobacteriaceae* of 3 × 10^2^ cfu/g. Presumptrive *Salmonella* was detected in 68 (41%) and presumptive *Listeria* spp. in 106 (64%). Furthermore, in 8 (12%) of the 66 dry samples, yeasts and molds were detected.

In Lebanon, the lack of microbiological standards concerning the allowable load of microorganisms in pet food might be the cause of inadequate quality control, which in turn may be a potential health risk for both pets and their owners. The findings specify the need for the Lebanese authorities to monitor the microbiological quality of pet food. Moreover, this study contributes to the building of a database for knowledge development regarding the potential contamination of pet food by the abovementioned microorganisms.

## Data availability statement

The raw data supporting the conclusions of this article will be made available by the authors, without undue reservation.

## Author contributions

MS co-secured the funding and co-wrote the manuscript. MH co-conducted the laboratory work and co-wrote the manuscript. HD carried out the statistical analysis and co-wrote the manuscript. MD co-conducted the laboratory work and shared input in paper revision. HH conceptualized the project, co-secured the funding, and co-wrote the manuscript. All authors contributed to the article and approved the submitted version.

## Conflict of interest

The authors declare that the research was conducted in the absence of any commercial or financial relationships that could be construed as a potential conflict of interest.

## Publisher's note

All claims expressed in this article are solely those of the authors and do not necessarily represent those of their affiliated organizations, or those of the publisher, the editors and the reviewers. Any product that may be evaluated in this article, or claim that may be made by its manufacturer, is not guaranteed or endorsed by the publisher.
